# Total thoracoscopic repair of ventricular septal defect: A single‐center experience

**DOI:** 10.1111/jocs.15504

**Published:** 2021-03-30

**Authors:** Kan Zhou, Liang Yang, Biao‐Chuan He, Ying‐Jie Ke, Yan‐Chen Yang, Qian Yan, Ze‐Rui Chen, Huan‐Lei Huang

**Affiliations:** ^1^ Guangdong Provincial People's Hospital (Guangdong Academy of Medical Sciences) Guangzhou Guangdong Province China; ^2^ Department of Cardiovascular Surgery Guangdong Provincial Cardiovascular Institute Guangzhou China; ^3^ The Second School of Clinical Medicine Southern Medical University Guangzhou China; ^4^ Nanhai Hospital of Guangdong Provincial People's Hospital Guangdong China

**Keywords:** mini‐sternotomy, total thoracoscopic repair, ventricular septal defect

## Abstract

**Objectives:**

To explore the safety and efficacy of total thoracoscopic repair of ventricular septal defects (VSD). We compared clinical outcomes of VSD via a total thoracoscopic approach with those of mini‐sternotomy.

**Methods:**

We retrospectively reviewed clinical data from patients with VSD from 2012 to January 2019. According to the surgical pattern, they were divided into two groups: the total thoracoscopic surgery group (36 patients, 27 females, aged 29 ± 9.52 years), and a mini‐sternotomy group (31 patients, 12 females, aged 28 ± 8.67 years).

**Results:**

There were no deaths in either group. In the thoracoscopic group, cardiopulmonary bypass (CPB) time and aortic cross‐clamping (ACC) time were significantly longer than those of the mini‐sternotomy group (CPB time: 112 ± 23.16 min vs. 78 ± 37.90 min, respectively, *p* < .001; ACC time: 65 ± 19.94 min vs. 50 ± 24.90 min, respectively, *p* < .001). postoperative hospital stay time (5.11 ± 2.48 days vs. 5.90 ± 6.27 days, *p* = .488) and chest drainage (139.86 ± 111.71 ml vs. 196.13 ± 147.34 ml, *p* = .081) tended to be lower in the thoracoscopy group, although there was no significant difference. No residual shunt or tricuspid regurgitation was found at follow‐up.

**Conclusions:**

Total thoracoscopic repair is safe and effective in patients with VSD, with or without tricuspid regurgitation.

Ventricular septal defects (VSD) are one of the most common congenital cardiac defects apart from atrial septal defect (ASD). Since the first VSD was performed by Lillehei in 1954,^1^ surgical repair of VSD via conventional sternotomy has become increasingly common, with low morbidity and mortality. Because of the need for favorable cosmetic effects, most adult patients, especially females, seek minimally invasive approaches for surgical repair. Currently, minimally invasive surgical options consist of mini‐sternotomy, right mini‐thoracotomy, and totally thoracoscopic repair for adult VSD.^2^, ^3^, ^4^ Of these, totally thoracoscopic repair via small port accesses in the right chest wall minimizes surgical trauma to the greatest extent.

Nevertheless, in China, due to conservative tendencies and insufficient publicity regarding improved cosmesis, there are worries regarding the costs of increased cardiopulmonary bypass (CPB) duration and possible worse patient outcomes, even though total thoracoscopic repair has shown high rates of successful repair, low morbidity and decreased length of stay in hospital.^5^ Therefore, in the present study, we explored the safety and efficacy of total thoracoscopic repair of VSDs, and compared the clinical outcomes of total thoracoscopic with those of mini‐sternotomy.

## 1 PATIENTS AND METHODS

### 1.1 Patient selection

We retrospectively reviewed the clinical outcomes of consecutive patients who underwent minimally invasive VSD patch repair at our institution between 2012 and 2019. The data were collected in our departmental database. The selection criteria for totally thoracoscopic VSD repair at our department were as follows: (1) perimembranous, membranous or inlet VSD as diagnosed by preoperative echocardiography; (2) body weight ≥30 kg; (3) no concomitant thoracic deformity or history of surgery of the right chest; (4) no concomitant femoral artery or aortic malformation, severe aortic atherosclerosis, or other cardiac malformations (patent ductus arteriosus, persistent left superior vena cava [SVC]); and (5) no concomitant severe pulmonary artery systolic pressure.

The outcomes of patients who underwent VSD repair by total thoracoscopic were compared with those of patients who underwent mini‐sternotomy. The outcomes of interest included hospital mortality, ventilation time, intensive care unit (ICU) stay time, postoperative hospital time, rates of blood transfusion, volume of chest drainage and postoperative complications. The postoperative complications included low cardiac output syndrome, respiratory failure, stroke, myocardial infarction, reoperation for bleeding, tricuspid regurgitation, residual shunt, complete atrioventricular conduction block, and wound infections. The requirement for individual patient consent was waived in light of the retrospective nature of the database assessment.

## 2 SURGICAL TECHNIQUES

### 2.1 Total thoracoscopic approach

**Figure 1:**
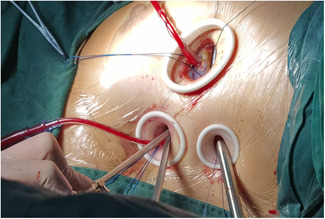
Three ports. The first port (2.5–3 cm) was positioned in the fourth intercostal space outside the right midclavicular line. The second port (1–1.5 cm) was placed in the fourth intercostal space anterior axillary line. The third port (1.0–2.0 cm) was located in the fifth intercostal space between the midaxillary line and anterior axillary line. A tissue retractor was insert into the port immediately if each port was made

**Figure 2:**
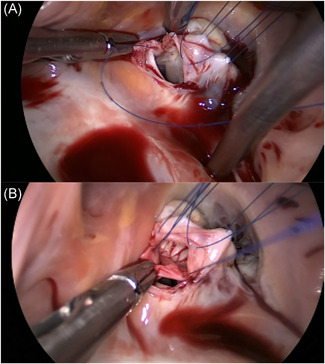
(A) Detachment of septal leaflet of tricuspid valve to expose the VSD. The septal tricuspid valve was partially detached by a circumferential parallel incision 2 mm away from the annulus, and the septal leaflet was suspended by 5‐0 Prolene. (B) Ventricular septal defect repair using a bovine patch. VSD, ventricular septal defects

Patients in the thoracoscopic group (TT) were positioned supine in a 15–20 degrees left lateral decubitus position. Double lumen endotracheal tube intubation with a transient single lung ventilation strategy was performed under general anesthesia, and then venous cannulation consisted of a right percutaneous (16‐Fr) SVC drainage catheter placed through the internal jugular vein, using ultrasonography guidance for placement. Three small ports were made on the right side of the chest. The first port (2.5–3 cm) was positioned in the fourth intercostal space outside the right midclavicular line. This port was used for the insertion of surgical instruments such as acutenaculums and scissors. The second port (1–1.5 cm) for the entry of instruments was handled by the left hand of the operator. The cross‐clamp of aorta (ACC) occlusion forceps was made in the fourth intercostal space, anterior axillary line. The third port (1.0–2.0 cm) for the placement of the 5‐mm thoracoscope was located in the fifth intercostal space between the midaxillary line and the anterior axillary line (Figure 1). A tissue retractor was inserted into the port immediately if each port was made. This could fix the incision open to protect the muscle and intercostal vessels, while facilitating access of the scope and instruments. The right common femoral vein was cannulated with a multiport (24‐ or 28‐Fr) venous drainage catheter. The vena cava were isolated with separate tourniquet snares, similar to sternotomy‐based surgery. The right common femoral artery was cannulated using a 17‐ or 19‐Fr arterial cannula. The ascending aorta was cross‐clamped with a transthoracic aortic cross‐clamp and antegrade cardioplegia was delivered into the aortic root, while the body temperature dropped to 32°C. A midbody right atriotomy was made after the superior and inferior vena cava were blocked. If the VSD could be exposed directly, it was closed with a patch of autologous pericardium or a bovine patch. If the VSD was inadequately exposed using the transatrial approach, detachment of the tricuspid valve was performed. The septal tricuspid valve was partially detached by a circumferential parallel incision 2 mm away from the annulus, and the septal leaflet was suspended by 3 or 4 sutures (Figure 2). After the VSD was continuously sutured with a patch, the septal leaflet was reattached to the annulus with a continuous suture, with the patch sandwiched between the leaflet and the annulus. Finally, the tricuspid valve coaptation and competence were assessed by injecting the cold saline into the right ventricle. The right thoracic cavity was flooded with CO_2_ via the second port throughout the procedure to avoid gas embolisms. Transesophageal echocardiography was used in each patient immediately after the VSD repair.

### 2.2 Mini‐sternotomy approach

In the mini‐sternotomy group, patients underwent a partial midline sternotomy for surgical exposure.^6^ Extracorporeal circulation was applied with central arterial and venous cannulation. The myocardial protection and the VSD repair were similar to that performed in the conventional sternotomy procedure. Transesophageal echocardiography was also used in each patient.

### 2.3 Statistical analysis

The data were prospectively collected in a computerized database, and then analyzed by Statistical Package for Social Sciences, version 22.0 (SPSS Inc.). All continuous variables were expressed as the mean ± *SD*s if normally distributed, otherwise median and interquartile. And the differences were tested for significance using the Student *t* test if normally distributed, otherwise Mann–Whitney's *U* test. The differences in categorical variables were tested for significance with the *χ* ^2^ or Fisher exact test, as appropriate, and were presented as proportions unless stated otherwise. The *p* < .05 was considered to be statistically significant in results.

## 3 RESULTS

During the study period, 67 patients underwent minimally invasive VSD repair at our institution, of whom 36 underwent total thoracoscopic repair and 31 underwent mini‐sternotomy repair. There was no significant difference in basic preoperative data between the groups, except for the higher proportion of women in the total thoracoscope group (75% vs. 38.7%; *p* = .003). Preoperative echocardiography showed 33 perimembranous VSDs and three inlet VSDs in total thoracoscopic group, while four perimembranous VSDs and 27 doubly committed and juxta‐arterial VSD. See Table 1 for details.

**Table 1:** Preoperative patient data

Variables	TT (*n* = 36)	MS (*n* = 31)	*p* Value
Female, *n* (%)	27 (75)	12 (38.7)	.003
Age, years	29 ± 9.52	28 ± 8.67	.757
Weight, kg	56 ± 9.08	58 ± 8.68	.413
NYHA class ≥III	0	2 (6.5)	.210
LVEF (%)	67 ± 4.43	67 ± 5.12	.634
Smoking, *n* (%)	2 (5.5)	3 (9.7)	.862
Comorbidities			
Hypertension, *n* (%)	1 (2.7)	2 (6.5)	.894
Diabetes mellitus, *n* (%)	2 (5.5)	1 (3.2)	1.000
CAD, *n* (%)	0	0	NA
PAD, *n* (%)	0	1 (3.2)	.940
COPD, *n* (%)	0	1 (3.2)	.940

*Note*: Data presented as mean ± *SD* or *n* (%).

Abbreviations: BMI, body mass index; CAD, coronary artery disease; COPD, chronic obstructive pulmonary disease; LVEF, left ventricular ejection fraction; MS, mini‐sternotomy group; NYHA, New York Heart Association; PAD, peripheral artery disease; TT, total thoracoscopic surgery group.

All patients underwent surgery on an elective basis. The mean CPB and ACC time in the total thoracoscopy group was significantly longer than that of the mini‐sternotomy group. Five patients in the total thoracoscopy group underwent tricuspid valvuloplasty with Edward ring followed by VSD repair. They suffered from tricuspid regurgitation due to annular or right ventricular dilatation as a result of left‐to‐right shunt. In 24 patients, for whom exposure of VSD was difficult due to septal leaflets attached to the rim of the VSD to form an aneurysm or abnormal chordal attached to the septum, underwent tricuspid valve detachment in the total thoracoscopy group. They were all free from atrioventricular conduction block and tricuspid regurgitation during the follow‐up period. See Table 2 for the relevant data.

**Table 2:** Surgical characteristics

Variables	TT (*n* = 36)	MS (*n* = 31)	*p* Value
CPB time, min	112 ± 23.16	78 ± 37.90	<.001
ACC, min	65 ± 19.94	50 ± 24.90	<.001
TVD, *n* (%)	24 (66.7)	0	<.001
TVP, *n* (%)	5 (13.9)	0	.057

*Note*: Data presented as mean ± *SD* or *n* (%).

Abbreviations: ACC, aortic cross‐clamp; CPB, cardiopulmonary bypass; MS, mini‐sternotomy group; NA, not applicable; TT, total thoracoscopic surgery group; TVD, detachment of septal leaflet of tricuspid valve; TVP, tricuspid valvuloplasty.

There were no inpatient deaths in either group (Table 3); however, one patient in the mini‐sternotomy group underwent tracheotomy due to pulmonary infection and could not be taken off the ventilator. Finally, the patient was discharged from the hospital and transferred to a local hospital for further treatment. The patient did not die during follow‐up. Only one patient in the TT group was found to have residual shunt immediately after coming off bypass, measured at 1.5 mm using transesophageal echocardiography. It disappeared spontaneously by the time of discharge. No patients underwent reoperation for bleeding in the TT group; however, one patient did so in the mini‐sternotomy group. This patient had bleeding because of the sternotomy. Postoperative hospital stay time (5.11 ± 2.48 days vs. 5.90 ± 6.27 days, *p* = .488) tended to be shorter in the thoracoscopy group than in the mini‐sternotomy group, suggesting faster recovery in the thoracoscopy group. In addition, the chest drainage was less in the thoracoscopy group than in the mini‐sternotomy group (139.86 ± 111.71 ml vs. 196.13 ± 147.34 ml, *p* = .081). One patient in the mini‐sternotomy group had wound infection, which recovered after wound debridement and vacuum sealing drainage. There were no low cardiac output syndromes, strokes, complete atrioventricular conduction blocks, myocardial infarction, or tricuspid regurgitation in either group. Follow‐up duration ranged from 12 to 96 months. No patient died, and echocardiography showed no residual shunts or tricuspid regurgitation at follow‐up.

**Table 3:** Early mortality and complications

Variables	TT (*n* = 36)	MS (*n* = 31)	*p* Value
Hospital mortality, *n* (%)	0	0	NA
Ventilation time, h	6 (4, 7.75)	6 (5, 9)	.443
ICU stay, h	21 (16.25, 22)	20 (16, 22)	.635
Postoperative hospital stay, days	5.11 ± 2.48	5.90 ± 6.27	.488
Rate of blood transfusion, *n* (%)	2 (5.5)	5 (16.1)	.312
Volume of chest drainage, ml	139.86 ± 111.71	196.13 ± 147.34	.081
Complication, *n* (%)			
LCOS	0	0	NA
Respiratory failure	0	1 (3.2)	.940
Stroke	0	0	NA
Myocardial infarction	0	0	NA
Reoperation for bleeding	0	1 (3.2)	.940
Tricuspid regurgitation	0	0	NA
Residual shunt	1 (2.7)	0	NA
CAVB	0	0	NA
Wound infection	0	1 (3.2)	.940

*Note*: Data presented as mean ± *SD* or *n* (%).

Abbreviations; CAVB, complete atrioventricular conduction block; ICU, intensive care unit; LCOS, low cardiac output syndrome; MS, mini‐sternotomy group; NA, not applicable; TT, total thoracoscopic surgery group; TVP, tricuspid valvuloplasty.

## 4 DISCUSSION

VSD is one of the most common congenital heart diseases, accounting for up to 40% of all congenital cardiac malformations.^5^ Because of fetal heart color Doppler ultrasound screening and heart murmurs after birth, most VSDs are detected at birth and are treated in infancy and early childhood. Only a small percentage of these patients are retested in adulthood, either because VSD was not detected at birth as a result of relatively low screening levels for congenital cardiac disease decades ago, or because surgery was delayed for financial reasons. Because of most patients being asymptomatic, these patients were found to have VSDs in the course of college entrance examinations or orientation medical examination in China. With the rapid development of endoscopic technology over the past 10 years, increasing numbers of patients begin to choose this minimally invasive technique. In addition, compared with conventional surgery and thoracotomy, total thoracoscopic technology is favored by patients because there is no metal implant and there is not much trouble on physical examinations at the company or security check at airports or railway stations. Results of conventional surgical VSD patch closure are excellent with low operative mortality and morbidity. Patients undergoing VSD repair are younger and relatively healthier than other patients undergoing cardiac surgery, and they are interested in more cosmetically appealing incisions. Nevertheless, they doubt that the less invasive approach provides cosmesis at the expense of the excellent outcomes typical of conventional surgery.^7^

The total thoracoscopic technique, with or without robotic surgery, has been widely used for ASD repair, VSD repair, mitral valve repair or replacement, tricuspid valve repair or replacement, ablation of atrial fibrillation, resection of cardiac myxoma, and even coronary bypass grafting.^8^, ^9^, ^10^, ^11^, ^12^, ^13^ However, in China, because robots are expensive, only a handful of hospitals have robots to perform surgery. Chinese surgeons tried to perform total thoracoscopic procedures without the aid of robots. At present, almost all clinical studies have shown that the mortality and complication rates of total thoracoscopic surgery are not inferior to those of median thoracotomy, and the former affords less bleeding, faster recovery and less trauma. Our institution's comparative outcomes also support the conclusion that total thoracoscopic VSD repair results in similar excellent results as those of mini‐sternotomy. Although the mean CPB and ACC time in the total thoracoscope group were significantly longer than those of the mini‐sternotomy group, few postoperative complications were suffered in the thoracoscopy group. Although the difference in tracheal intubation time, ICU time, postoperative hospital‐stay time and chest drainage did not reach statistical significance, these variables tended to be lower in the thoracoscopy group, which possibly demonstrating the superiority of less bleeding, faster recovery and less trauma.

Our institution believes that the perimembranous, membranous or inlet VSD are more suitable for total thoracoscopic surgery. If pouch formation of the septal leaflet of the tricuspid valve or multiple chordae tendineae cross over the defect, the detachment of septal leaflet of tricuspid valve is performed to expose these VSDs. Perhaps traditionalists remain concerned that detachment may increase the incidence of iatrogenic complications such as atrioventricular conduction block and tricuspid valve insufficiency. However, tricuspid valve detachment has been previously shown excellent outcomes.^14^, ^15^, ^16^, ^17^ Our results also suggest the detachment was a safe and effective technique. Twenty‐four patients underwent tricuspid valve detachment in our study and none showed atrioventricular conduction block or tricuspid regurgitation. By contrast, outlet VSD are difficult to expose in the visual field of the thoracoscopic approach, and the surgical instruments are usually not long enough because of the depth of the thorax in adults. Muscular VSDs are usually situated near the apex and often have many outlets on the right ventricular side. As a result, if thoracoscopy is used, residual shunt is more likely to occur, so muscular VSD are more suitable interventions.^18^, ^19^

Total thoracoscopic surgery is not available for all patients. If patients have concomitant thoracic deformities, pleural adhesions, femoral artery or aortic malformations, severe aortic atherosclerosis, or other cardiac malformations (patent ductus arteriosus, persistent left SVC), total thoracoscopic surgery is not appropriate. At our institution, if thoracoscopic surgery is required, thoracic computed tomography (CT) and total aortic CT should be performed before surgery to exclude these concomitant diseases, so as to avoid intraoperative transition to surgery due to severe pleural adhesions, or failure to perform peripheral femoral arteriovenous cannulation due to femoral artery malformation, or aortic dissection due to severe aortic atherosclerosis.

Of course, total thoracoscopic surgery is not without its disadvantages and limitations. Although total thoracoscopy reduces the incision and trauma in the chest, it increases use of neck and groin vessels, thereby increasing the risk of peripheral nerve or vessel injuries such as femoral arteriovenous stenosis, femoral arteriovenous fistula, femoral nerve injury, and jugular arteriovenous fistula. Attention should be paid to vascular dissociation and vascular puncture and intubation operation to avoid injury. The intubation operation should be soft, and if the operation run into resistance, forced insertion should not be carried out to avoid vascular injury or even femoral artery dissection. There was one case of retrograde aortic dissection caused by femoral artery cannulation in our center in the early stages of total thoracoscopic surgery. In addition, due to the need for double lumen endotracheal tube intubation with a transient single lung ventilation strategy in total thoracoscopic surgery, postoperative atelectasis or pneumothorax often occurs.

## 5 LIMITATIONS

This study was subject to the typical limitations of a retrospective case series. There were several limitations, including small sample size and differences in the patient populations. Most patients underwent VSD repair in infancy and early childhood, and we excluded patients with severe pulmonary arteria systolic pressure and other concomitant diseases mentioned in the discussion section. As a result, the sample size was small. In addition, while most preoperative characteristics were well matched, there were significant differences in sex and VSD types among the two groups. More females opted for the total thoracoscopic procedure due to the need for favorable cosmesis. We excluded the subarterial or supracristal VSDs in the thoracoscopy group, which led to the vast majority proportion of this type being in the mini‐sternotomy group.

## 6 CONCLUSION

VSD repair can be performed safely and effectively via total thoracoscopy with excellent outcomes similar to those of ministernotomy.

## CONFLICT OF INTERESTS

The authors declare that there are no conflict of interests.

## Data Availability

All the data are available from the corresponding author.
